# Knowledge, Attitudes, and Practices Toward Self-Medication Among Pharmacy Undergraduates in Penang, Malaysia: A Cross-Sectional Study

**DOI:** 10.3390/pharmacy13030079

**Published:** 2025-06-02

**Authors:** Bayan F. Ababneh, Hisham Z. Aljamal, Rabia Hussain

**Affiliations:** 1 Discipline of Social and Administrative Pharmacy, School of Pharmaceutical Sciences, Universiti Sains Malaysia, Gelugor Penang 11800, Malaysia; bayanfabab@hotmail.com; 2 Discipline of Orthopedics, Dr. Sulaiman AL Habib Hospital, Al Khobar 34236, Saudi Arabia; hishamzjamal@gmail.com

**Keywords:** self-medication, knowledge, attitude, practices, prevalence, pharmacy students, Malaysia

## Abstract

Background: Self-medication is the use of medicinal products to treat self-diagnosed disorders or symptoms without the prescription or supervision of a healthcare professional. There is a lack of data about self-medication knowledge, attitudes, and practices among pharmacy undergraduates in Malaysia. This study assessed the knowledge, attitudes, and practices among undergraduate pharmacy students in Penang regarding self-medication. Method: A descriptive cross-sectional study was conducted using a self-administered, web-based survey (Google Forms), which was completed and responded to by 203 undergraduate pharmacy students from Penang, Malaysia, between October and December 2023. Descriptive statistics were used to summarize the socio-demographic characteristics of the participants. Associations between the socio-demographic characteristics of the participants and the knowledge, attitudes, and practices regarding self-medication were assessed using a chi-square test. Regression analyses were carried out to determine whether the socio-demographic characteristics of the participants were associated with practices of self-medication. Results: A total of 203 of the undergraduate pharmacy students completed the questionnaire. More than half of the participants’ age ranged between 19 and 21 years old, the majority were females (77.3%), and 31.5% of the participants had family members employed in the healthcare sector. Most respondents showed good knowledge in a variety of domains: 97.5% acknowledged the potential for drug interaction with other medications, indicating a high awareness of proper self-medication practices. A positive attitude was found regarding participants’ attitudes toward self-medication, and 65.5% practiced self-medication, primarily for treating minor illnesses (75.9%). Common conditions included fever (83.3%), cough/cold/flu (76.8%), and headache (71.4%). Reasons for not self-medicating included the absence of illness (20.2%), lack of knowledge/prior experience (19.2%), and fear of using the wrong medication (18.7%). Only academic year level was the predictor of practicing self-medication within the last six months among the participants. Conclusions: Generally, the participants possessed good knowledge and positive attitudes toward self-medication. The study revealed no significant associations between demographic characteristics and knowledge or attitudes. Insights from this research contribute to understanding self-medication practices among pharmacy students in Penang, informing potential interventions to promote responsible self-medication practices.

## 1. Introduction

According to the World Health Organization (WHO), self-medication involves the use of medicinal products by the consumer to treat self-diagnosed disorders or symptoms or the intermittent or continued use of medication prescribed by a physician for chronic or recurrent diseases or symptoms [1]. It involves the use of medications, herbs, or home remedies based on one’s initiative or the advice of others without the prescription or supervision of a healthcare professional [2]. Using over-the-counter medications for self-diagnosis, sharing prescriptions with friends or family, and taking medications left over from previous illnesses at random are all considered forms of self-medication [3].

This practice is a global phenomenon and is prevalent in both developed and developing countries [4]. The highest incidence rate of self-medication was observed in Europe (Eastern) (74%, 95% CI, 56–86%) and Asia, with a prevalence of 71% (95% CI, 63–78%). In terms of the ailment that prompted self-medication, neurological conditions accounted for 48% of the patients’ self-medication behaviors (95% CI, 40–55%). “A prior history” and “the minor nature of the disease” were the most frequently cited causes of self-medication [4]. Self-medication typically has negative rather than positive benefits and might have long-term harmful consequences [5,6]. However, according to the WHO, proper self-medication is just as important as proper diet and personal hygiene as a component of self-care [3]. An international public health concern is the improper use of over-the-counter medications and their accessibility to those who lack enough knowledge and awareness [5,6,7].

Numerous variables, including socioeconomic circumstances, changing lifestyles, easy access to pharmaceuticals, the ineffectiveness of healthcare systems, high costs, inaccessibility, and uncontrolled drug distribution, have contributed to the rise in self-medication in recent years [8,9]. The advantages of this problem to consider include lower medical costs, fewer physician referrals, shorter wait times at medical facilities, and resource loss [7,10,11]. It may also be linked to negative consequences like drug dependence, drug resistance, possible delays in the timely diagnosis and treatment of serious health issues, concealing the disease’s latent symptoms and making it worse, illicit drug use, drug interactions, and unintended pharmaceutical effects [5,6,12].

Several studies have been conducted in this field that provide data on the prevalence of self-medication. Studies by Knopf et al. in Germany (2013) [13] and Garofalo et al. in Italy (2015) [14], for instance, revealed self-medication rates of 27.7% and 69.9%, respectively. In Iran (2015), Azami Aghdash et al. found that the prevalence of self-medication was 53% [14]. Ayalew et al. found that the rate of self-medication ranged from 12.8 to 77.1% in different Ethiopian studies [9].

Self-medication is more common among medical professionals and students who study health-related courses [15]. The prevalence of self-medication among pharmacy and or medical undergraduate students in different countries was reported [16,17,18,19,20,21,22,23,24,25,26,27,28,29]. A high prevalence of self-medication was found in Jordan among pharmacy students (82.9%), followed by Doctor of Pharmacy students (77.9%) [17]. The overall self-medication prevalence among Serbian pharmacy students was 81.3% [24]. Self-medication was quite prevalent among pharmacy students in Pakistan at 83.0% [25]. However, other countries reported a lower prevalence rate of self-medication among students, such as in Western Nepal, where it was 54% [16], and in India, where it was 42.2% [23].

In Malaysia, few studies explore self-medication among pharmacy or medical undergraduates [20,21,29]. The pharmacy students of International Islamic University Malaysia (IIUM) reported a prevalence of self-medication at 84% [20]. Another study reported a high prevalence of self-medication among pharmacy students at Universiti Kuala Lumpur Royal College of Medicine Perak (UniKL RCMP) at 99.10% [29]. Moreover, in University College in Malaysia, the prevalence of self-medication among dental and medical students was 57.11% [21].

Due to the limited number of studies conducted in Malaysia, comprehensive and consistent research on self-medication practices within the Malaysian context is lacking, particularly among pharmacy undergraduate students. Since pharmacy undergraduates play a vital role in the safe and efficient use of medications as future pharmacists, assessing their knowledge and attitudes toward self-medication is important. More research is required since the factors that influence self-medication behaviors are still under debate. Therefore, this study aimed to evaluate the knowledge, attitudes, and practices among undergraduate pharmacy students in Penang regarding self-medication, including their understanding of appropriate practices, drug interactions, and contraindications; to measure the attitude toward self-medication; and to investigate the actual self-medication practices of students, including the prevalence, frequency, and reasons for engaging in self-medication. Moreover, socio-demographic factors affecting the knowledge, attitudes, and practices of self-medication were explored.

## 2. Materials and Methods

### 2.1. Research Design and Setting

A descriptive cross-sectional study was employed to evaluate knowledge, attitudes, and practices toward self-medication. A self-administered survey using an online Google Form was utilized as a data-collecting tool and was optimized to be easily filled out using computers and smartphones. The survey was distributed online over two months from October to December 2023, via the Google Forms platform. A non-probability convenience sampling method was used for data collection. The study population comprised undergraduate pharmacy students in Penang, Malaysia.

### 2.2. Participants

The sample size was calculated using Cochran’s sample size formula for small populations with a known size n = n_0/(1 + (n_0−1)/N) [30]. The total population for this study comprised 458 undergraduate pharmacy students in Penang, spanning Year 1 to Year 4 of the 2023/2024 academic year. According to a study conducted at University College in Malaysia, the prevalence of self-medication was found to be 57.1% [21]. Based on that study, the *p*-value was 0.57, and a 95% confidence interval and ±5% precision were used. So, according to Cochran’s formula with smaller sample correction, the recommended size for this study was 208 participants [30].

Pharmacy students from all academic years (Year 1, Year 2, Year 3, and Year 4) who provided informed consent were eligible to participate in this study.

### 2.3. Research Instrument

To evaluate the knowledge, attitudes, and practices among undergraduate pharmacy students in Penang regarding self-medication, based on extensive literature, a closed-ended questionnaire (Supplementary Material, Table S1) was designed, validated, and presented in the English language [20,21,22,27,31,32,33].

This questionnaire underwent content validation by an evaluation team comprising three academics from clinical pharmacy and pharmacy practice backgrounds before distribution to the participants. Any amendments were made based on the feedback and suggestions received. A content validity form was developed with a five-point Likert scale and emailed to the experts. They were requested to determine whether all items referred to the relevant aspects of constructs to be measured (1 = relevant, 5 = irrelevant), the importance of each item (1 = essential, 5 = not necessary), and any items missing in the questionnaire. To ensure the face validity of this study, a pilot study was conducted with 22 undergraduate pharmacy students who were selected conveniently and excluded from the final analysis to test the instrument before being distributed to the participants so that the reviewed questionnaire was properly designed and understandable for the study population [34]. To ensure the reliability of the instrument, internal consistency, which measures how closely related the items are for each scale, was calculated using Cronbach’s alpha coefficient for the perception score, with values of 0.70 and above indicating good internal consistency [35].

This questionnaire was divided into four sections with thirty-seven items. This included the socio-demographic questions (8 items), participants’ knowledge of self-medication (10 items), attitudes toward self-medication (10 items), and practices of self-medication (9 items).

The knowledge, attitudes, and prevalence of self-medication practices among the participants were measured using Bloom’s cutoff point.

For the second section (knowledge), correct answers were coded as “1”, while incorrect answers were coded as “0”. The maximum score was 10, and the minimum was 0. If participants obtained 8–10 points, they were considered to have good knowledge, 6–7 points were considered to indicate moderate knowledge, and less than 6 points was considered to indicate poor knowledge of self-medication [23].

The third section (attitude) was presented in positive statements that came together on a Likert scale, from strongly agree [5], agree [4], neutral [3], disagree [2] to strongly disagree [1]. The score was categorized using the modified Bloom’s cutoff point. The maximum score was 50, and the minimum was 10. If the participant obtained 35–50 points, they were categorized as having a positive attitude, 16–34 points indicated a neutral attitude, and 0–15 points indicated a negative attitude toward self-medication practices [23].

For the fourth section (practices), the frequencies of selected answers from participants were calculated.

### 2.4. Ethical Consideration

Ethics approval for this study was obtained from the Joint Ethics Committee on Clinical Studies of the School of Pharmaceutical Sciences, USM, and Hospital Lam Wah Ee (USM-HLWE/IEC/2023 (0006)/exempt). Moreover, the participants who agreed to take part in the study signed the consent form electronically before proceeding to the first section of the instrument. Participation in this study was voluntary, and participants had the right to withdraw at any time, and no incentives were provided.

### 2.5. Statistical Analysis

Descriptive statistics were used to summarize the socio-demographic characteristics of participants. Categorical variables data were presented as frequency and percentage. To assess the normality, the Kolmogorov–Smirnov test was performed. Since the data did not support parametric assumptions, the median and interquartile range were reported for continuous data. Bivariate analysis, such as a chi-square test, was performed to assess the association of demographic characteristics and the practice of self-medication, with good associations (*p* < 0.2), which was included in the multivariate logistic regression. For the logistic regression model, the first category of each independent categorical variable was used as the reference category. We assessed the model’s goodness-of-fit using the Hosmer–Lemeshow test. Odds ratios (ORs) with 95% CI were calculated to estimate the strength of associations. The internal consistency of the instrument scales was assessed using Cronbach’s alpha. A *p*-value of 0.05 was used for all statistical tests, which were two-tailed. Analyses were performed using the IBM statistical package for social science (IBM SPSS) version 28 (SPSS Inc., Chicago, IL, USA).

## 3. Results

### 3.1. Socio-Demographic Characteristics of the Participants

Two hundred and three questionnaires were distributed to the undergraduate pharmacy students in Penang. A total of 203 of the undergraduate pharmacy students completed the questionnaire. The age of more than half of the participants ranged between 19 and 21 years. Briefly, the majority were females (77.3%, *n* = 157) and from the second academic year (28.1%, *n* = 57), and all were single and full-time students. Additionally, 73.9% (*n* = 150) of the participants resided in university housing, and 31.5% (*n* = 64) of the participants had family members employed in the healthcare sector. Table 1 summarizes the socio-demographic data of all participants.

### 3.2. Reliability and Validity of the Study Instrument

The face and content validity of the study instrument were satisfactory, as found by the expert panel. The Cronbach’s alpha coefficient for the study instrument was 0.782, which shows that the data exhibited strong internal consistency in the format used.

### 3.3. Knowledge of Self-Medication

Table 2 represents the knowledge of self-medication among the participants. The median knowledge score of the participants was 9, with an interquartile range (IQR) of 1. Most respondents showed good knowledge in a variety of domains: 97.5% (*n* = 198) of the participants acknowledged the potential for drug interaction with other medications and alcoholic drinks, while 95.6% (*n* = 194) recognized the influence of certain foods on drug efficacy. Moreover, the majority showed good knowledge about contraindications among specific populations, including children (97.5%) (*n* = 198), pregnant (98.0%) (*n* = 199) or breastfeeding individuals (97.5%) (*n* = 198), and those with chronic illness (96.1%) (*n* = 195). However, 51.7% (*n* = 105) noticed some areas of improvement that cause potential issues with adherence, admitting to stopping medication without professional consultation. Despite this, 88.2% (*n* = 179) recognized the risk associated with medication sharing, and 93.1% (*n* = 189) claimed that they checked the medications’ expiry date before purchasing.

#### Factors Associated with Participants’ Knowledge of Self-Medication

Table 3 demonstrates the factors associated with knowledge of self-medication. The chi-square association test indicates that there were no statistically significant relationships (*p* > 0.05) between participants’ self-medication knowledge levels and demographic characteristics.

### 3.4. Attitudes Toward Self-Medication

A positive attitude was found regarding participants’ attitudes toward self-medication (Table 4). The median attitude score of the participants was 31.0, with an interquartile range (IQR) of 7.1. The majority of participants (40.9%, *n* = 83) believed that self-medication is a part of their self-care. Only 20.2% (*n* = 41) and 16.7% (*n* = 34) of the participants recommended self-medication to themselves and others, respectively. Additionally, 40.9% (*n* = 83) of participants believed in the necessity of training for self-medication practice, while 20.7% (*n* = 42) attributed the main cause of self-medication to be accessibility to healthcare information.

#### Factors Associated with Participants’ Attitudes Toward Self-Medication

Table 5 explores the factors associated with participants’ attitudes toward self-medication. Factors are distributed into their different attitude: positive, neutral, and negative. The chi-square association test indicates that there were no statistically significant relationships (*p* > 0.05) between participants’ self-medication attitude levels and demographic characteristics.

### 3.5. Practice of Self-Medication

The students’ self-medication practices are described in Table 6. About two-thirds (65.5%, *n* = 133) of the participants had practiced self-medication, and 26.1% (*n* = 53) reported practicing self-medication during the last month, while 23.2% (*n* = 47) practiced self-medication during the last three months. However, 34.5% (*n* = 70) reported not practicing any self-medication due to fear of using the wrong medication (18.7%, *n* = 38) and lack of knowledge and experience (19.2%, *n* = 39).

The main sources of information about self-medication among the participants were experiences from previous treatments (51.7%, *n* = 105) and healthcare professionals (49.3%, *n* = 100). About half of the participants claimed to obtain their medication for self-medication purposes through retail community pharmacies (65.0%, *n* = 132), and the majority of them requested medication by either mentioning the name of the medication (82.2%, *n* = 167) or the signs and symptoms of illness (77.3%, *n* = 157).

**Figure 1:**
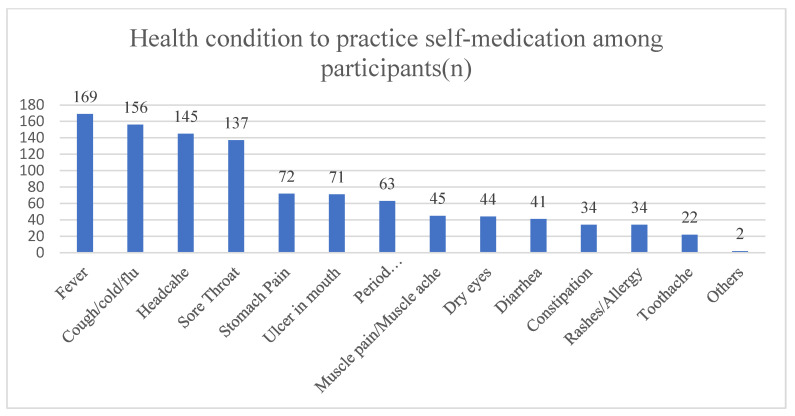
Health condition to practice self-medication among participants.

**Figure 2:**
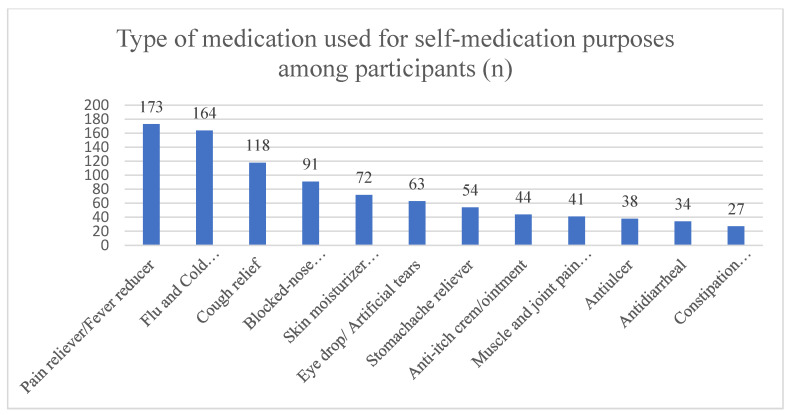
Type of medication used for self-medication purposes among participants.

Among the most frequent health conditions that led the participants to practice self-medication were fever (83.3%, *n* = 169) and cough/cold/flu (76.8%, *n* = 156), followed by headache (71.4%, *n* = 145) and sore throat (67.5%, *n* = 137) (Figure 1). Both pain reliever/fever reducers (85.2%, *n* = 173) and flu and cold relief/allergy medication (80.1%, *n* = 164) were the most used medications for self-medication purposes, followed by cough relief medications (58.1%, *n* = 118) (Figure 2). Most of the participants claimed to have self-medicated due to the nonseriousness of the illness (75.9%, *n* = 154), and 62.6% (*n* = 127) had experience treating the same illness, followed by using time constraints (52.2%, *n* = 106).

#### Factors Associated with Participants’ Practices of Self-Medication

The relationship between the socio-demographic factors and participants’ practice of self-medication within the last six months is presented in Table 7. Only participants aged between 19 and 21 years (*p* = 0.027) and at the second academic year level (*p* = 0.001) were found to be associated with the practice of self-medication within the last six months.

Multiple logistic regression analysis shows that the participants’ educational level was the predictor of practicing self-medication within the last six months (Table 8).

## 4. Discussion

This study aimed to evaluate the knowledge, attitudes, and practices of undergraduate pharmacy students in Penang regarding self-medication. Overall, most participants (95.6%) exhibited good knowledge regarding the appropriate use of medication in self-medication. Additionally, 30.0% of the participants showed a positive attitude toward self-medication. The prevalence of self-medication in the past six months was found to be 65.5%.

### 4.1. Knowledge of Self-Medication

According to our study, most of the participants (95.6%) possessed a good knowledge of self-medication. The results are satisfactory, unlike other studies that showed a low level of knowledge regarding self-medication among university students in Portugal and Kuwait [36,37]. Pharmacy students were exposed to a diverse range of medications in their educational courses, which caused the contrast. The majority of the participants (93.1%) demonstrated high awareness of drug–drug interactions, drug–food interactions, contraindications between drugs and medical conditions, alerts on medicine expiry, and the importance of not sharing medication with others. This was consistent with the findings from other studies in Nepal, where more than half of the respondents had a high level of knowledge regarding self-medication [16,38]. Moreover, the majority (62.40%) of pharmacy students in Malaysia at UniKL RCMP showed good knowledge of self-medication [29]. However, the majority of pharmacy students showed poor knowledge of self-medication in Iran [39] and Malaysia at IIUM [20].

The most common sources of information about self-medication among participants were prior treatment experiences (28.5%) and references to healthcare professionals (27.1%). Approximately 48.7% of the participants reported obtaining their medication for self-medication purposes from retail community pharmacies. Through these resources, pharmacy students obtained their knowledge about diseases and medications. Hence, healthcare practitioners play a crucial role in providing valuable guidance on the appropriate and safe consumption of pharmaceutical medications. Pharmacy students must maintain a high level of awareness about the harmful consequences of irrational self-medication. A similar observation was reported among pharmacy and medical students in Saudi Arabia, where their source of information about self-medication was pharmacists and physicians [26].

Moreover, based on the results obtained, there was no significant (*p* > 0.05) association between any demographic characteristics and the level of knowledge of self-medication. However, these findings contradict those of studies conducted in Malaysia (UniKL RCMP), Portugal, Iran, and Saudi Arabia [18,29,36,39]. According to their research, sex and academic year substantially influence knowledge, with female students and higher academic year groups demonstrating a considerably greater level of knowledge. This discrepancy was probably due to the differences in the structure and curricula of pharmacy courses in Penang, Malaysia, compared to other international universities. The degree of knowledge among participant students was not affected by the academic year, suggesting that their undergraduate curricula comprehensively educate students about health knowledge throughout their academic years.

### 4.2. Attitudes Toward Self-Medication

A positive attitude was found regarding participants’ attitudes toward self-medication. The majority of participants (40.9%) believed that self-medication is a part of their self-care. Moreover, 40.9% of participants believed in the necessity of training for self-medication practice, while 20.7% attributed the main cause of self-medication to be accessibility to healthcare information. Similar findings were revealed by previously conducted studies. The undergraduate students in Riyadh and Dammam in the Kingdom of Saudi Arabia showed a positive attitude toward self-medication [26,27], and undergraduate nursing students at a private healthcare university college in Negeri Sembilan, Malaysia, demonstrated a positive attitude toward self-medication (92.2%) [33]. In contrast, the majority of pharmacy students (69.7%) at UniKL RCMP showed neutral attitudes toward self-medication [29]. In the present study, there was no significant association between the demographic characteristics and attitudes toward self-medication among participants. These findings contrast with those of studies conducted in Riyadh, where sex, study level, full/working student status, residential status, and marital status had significant impacts on attitudes [27]. As students progressed to higher study levels, a greater positive attitude toward self-medication was observed [26,27]. This discrepancy might be due to the variations in the structure and focus of pharmacy education programs between the institutions. The structure and focus of pharmacy education programs may influence students’ attitudes. The emphasis on self-medication education, coursework, and exposure to related topics could differ.

### 4.3. Practice of Self-Medication

The prevalence of self-medication in the past six months in this study was 65.5%. This corresponds with other studies. The prevalence of self-medication among pharmacy students in Nepal was 54% [16]. Moreover, in Egypt, the prevalence of self-medication was 62.9% [22]. A total of 42.2% of Indian pharmacy students documented the practice of self-medication [23]. The prevalence of self-medication among students in the UAE was 57.5% [19]. In Saudi Arabia, the prevalence of self-medication among different university students ranged between 49.3 and 87.0% [18,26,31,40]. In Manipal University College Malaysia, Melaka, the prevalence of self-medication among dental and medical students was 57.1% [21]. A higher prevalence of self-medication was documented among pharmacy students in both IIUM and UniKL RCMP at 84.0% and 99.1%, respectively [20,29].

The most common reason for the practice of self-medication documented by the participants was minor illness treatment (75.9%). Similar findings were found in a study conducted among medical and pharmacy students at Qassim University in Saudi Arabia, in which two-thirds (67%) of respondents reported that they practiced self-medication because of minor illnesses, followed by the need for quick relief (63.6%) [28]. Furthermore, 62.6% of the 127 respondents in this study reported practicing self-medication because they had used the medication before. Since they have prior experience with the medication, they tend to treat self-recognized minor ailments by themselves. Additionally, 52.2% of the 106 respondents reported indulging in self-medication due to time constraints. This corresponds with the results of a study conducted in Bahrain among medical students, where the most common reasons for self-medication were previous experience (45.5%), mild illness (40.3%), and a shortage of time (32.1%) [41]. Moreover, Indian students reported time constraints as the most common reason for self-medication practices [42]. Self-medication may be beneficial in facilitating the recovery of certain minor ailments. However, the risks and benefits must be weighed reasonably and assessed thoroughly because some adverse reactions may arise from the self-use of medication [43].

Among the most frequent health conditions that led the respondents to practice self-medication were fever (83.3%) and cough/cold/flu (76.8%), followed by headache (71.4%). Similar findings were reported in the literature, as most of the undergraduate medical students at Dow Medical College in Karachi, Pakistan (67.9%), reported that fever was the main and most frequent health condition that induced them to practice self-medication [25]. Additionally, more than half of the pharmacy students at Qassim University in Saudi Arabia (64.6%) [28] and Arabian Gulf University in Bahrain (70.9%) reported that headaches were the most frequent cause of self-medication practice [41]. Additionally, headaches (71.2%) and the common cold (56.5%) were the most common illnesses that led to self-medication among Jordanian pharmacy students [17].

The most reported medications for self-medication purposes in this study were pain relievers/fever reducers (85.2%) and flu and cold relief/allergy medication (80.1%). Similar results were reported in the literature in Portugal [36], Saudi Arabia [26], and Malaysia [20]. However, in Jordan, pharmacy students reported that analgesics (79.9%) and antibiotics (59.8%) were the most reported medications for self-medication [17]. The reason for this was that in some countries, antibiotics may be dispensed as over-the-counter medications. The use of antibiotics was not documented in the practice of self-medication in this study, indicating that pharmacy students in Penang have adequate knowledge about antimicrobial resistance and the consequences of the irrational use of antibiotics.

In this study, nearly two-thirds (65.5%) of the respondents reported practicing self-medication. Specifically, 26.1% reported practicing self-medication during the last month, and 23.2% reported it during the last three months. For the last six months, 33 (16.3%) had practiced self-medication. A total of 34.5% of the respondents reported not indulging or practicing any self-medication for several reasons. The most common reasons were absence of illness at a specified time (20.2%) and lack of knowledge and experience (19.2%), followed by fear of using the wrong medication (18.7%). A lack of knowledge and experience could create uncertainty and potential risks regarding self-medication. However, according to a study conducted by Imam Abdulrahman Bin Faisal University, Dammam, Saudi Arabia, the most frequent and common reason for not practicing self-medication was drug safety issues (38%) [26].

This study shows that only the academic year level as a demographic factor was associated with the participants’ practice of self-medication. During the second year of study, students appeared to self-medicate more than during later study years. Students become more educated and more knowledgeable about the topic as their academic careers continue. Professional training could have an impact on their self-medication practices. This finding is comparable to the studies conducted on students at Dammam University [26] and UniKL RCMP [29]. On the other hand, a significant relationship was observed between family members working in the healthcare sector and self-medication practice among students in Riyadh [27].

There are several limitations in the current study. Firstly, the instrument relied on participants’ self-rated assessment of their perceptions, which may have resulted in an overestimation of the results. This study was conducted at a single institution in Penang, which may limit the generalizability of the findings to the broader national context. Therefore, future research is recommended to include a wider range of institutions across different regions in order to enhance the representativeness and applicability of the results. Participants may not accurately recall details about their self-medication practices, as the study relies on participants’ memories of events or behaviors over a certain period. Nevertheless, this study reveals important information about the knowledge, attitudes, and practices of self-medication among pharmacy students. Last but not least, future studies could provide a deeper understanding of self-medication practices by comparing the knowledge, attitudes, and self-medication practices of pharmacy and other health sciences students from various institutions.

## 5. Conclusions

The study shows good knowledge and positive attitudes toward self-medication among pharmacy students in Penang. The documented prevalence practice of self-medication among pharmacy students in Penang was relatively lower than in UniKL RCMP and IIUM. Moreover, the most common reasons to practice self-medication were minor illnesses, previous experience with the medication, and time constraints. Fever, cough/cold/flu, and headache were prevalent conditions to practice self-medication. The study contributes valuable insights into self-medication practices among pharmacy students in Malaysia, urging future research to explore the impact of educational programs on attitudes and practices. Additionally, investigating pharmacy and other health sciences students from various universities, as well as healthcare professionals, would offer a more comprehensive understanding of self-medication practices.

## Figures and Tables

**Table 1:** Socio-demographic details of participants (N = 203).

Demographic Characteristics (N)		*n* (%)
Age (years)	19–21	149 (73.4)
	22–25	54 (26.6)
Sex	Male	46 (22.7)
	Female	157 (77.3)
Academic year	Year 1	50 (24.6)
	Year 2	57 (28.1)
	Year 3	52 (25.6)
	Year 4	44 (21.7)
Marital status	Single	203 (100.0)
Ethnicity	Malay	102 (50.2)
	Chinese	84 (41.4)
	Indian	9 (4.4)
	Other	8 (4.0)
Study status	Full-time student	203 (100.0)
Current residency	University housing	150 (73.9)
	Private housing	42 (20.7)
	With family	11 (5.4)
Family members working in the healthcare sector	Yes	64 (31.5)
	No	139 (68.5)

N: Sample size, *n*: frequency.

**Table 2:** Knowledge of self-medication among pharmacy undergraduates (N = 203).

Questions	Answered Correctly, *n* (%)
Do you know that some medications cannot be taken with other medications?	198 (97.5)
Do you know that some medications cannot be taken with alcoholic drinks?	198 (97.5)
Do you know that some medications cannot be taken with certain foods?	194 (95.6)
Do you know that some medications are contraindicated or cannot be given to children?	198 (97.5)
Do you know that some medications are contraindicated or cannot be given when pregnant?	199 (98.0)
Do you know that some medications are contraindicated or cannot be given when breastfeeding?	198 (97.5)
Do you know that some medications are contraindicated or cannot be given to people with chronic illnesses?	195 (96.1)
Did you stop taking your medications without consulting with a healthcare professional for confirmation or guidance?	105 (51.7)
Do you know that certain medications cannot be shared with family members, friends, neighbours, etc.?	179 (88.2)
Do you check the expiry date of the medications before purchasing/before use?	189 (93.1)
Knowledge score, median (IQR)	9.0 (1.0)

IQR: interquartile range, *n*: frequency, N: sample size.

**Table 3:** Factors associated with the participants’ knowledge about self-medication (N = 203).

Factors		Knowledge Level of Self-Medication			Chi-Square (x^2^)	*p*-Value
		Good, *n* (%)	Moderate, *n* (%)	Poor, *n* (%)		
Age	19–21	142 (95.3)	5 (3.4)	2 (1.34)	0.4	0.828
	22–25	52 (96.3)	1 (1.9)	1 (1.9)		
Sex	Male	43 (93.5)	2 (4.3)	1 (2.2)	0.6	0.736
	Female	151 (96.2)	4 (2.5)	2 (1.3)		
Academic year	Year 1	46 (92.0)	3 (6.0)	1 (2.0)	6.7	0.345
	Year 2	53 (93.0)	3 (5.3)	1 (1.6)		
	Year 3	52 (100.0)	0 (0.0)	0 (0.0)		
	Year 4	43 (97.7)	0 (0.0)	1 (2.3)		
Ethnicity	Malay	97 (95.1)	3 (2.9)	2 (2.0)	3.4	0.762
	Chinese	81 (96.4)	2 (2.4)	1 (1.2)		
	Indian	9 (100.0)	0 (0.0)	0 (0.0)		
	Other	7 (87.5)	1 (12.5)	0 (0.0)		
Current residency	University housing	143 (95.3)	6 (4.0)	1 (0.7)	7.4	0.117
	With family	10 (90.9)	0 (0.0)	1 (9.1)		
	Private housing	41 (97.6)	0 (0.0)	1 (2.4)		
Family members working in the healthcare sector	Yes	60 (93.75)	3 (4.7)	1 (1.6)	1.0	0.611
	No	134 (96.4)	3 (2.2)	2 (1.4)		

*n*: frequency, N: sample size.

**Table 4:** Attitudes toward self-medication among pharmacy undergraduates (N = 203).

Statements	Strongly Agree *n* (%)	Agree *n* (%)	Neutral *n* (%)	Disagree *n* (%)	Strongly Disagree *n* (%)
I believe self-medication is a part of self-care.	83 (40.9)	73 (36.0)	32 (15.8)	12 (5.9)	3 (1.5)
I would like to start/continue my self-medication therapy.	41 (20.2)	70 (34.5)	59 (29.1)	22 (10.8)	11 (5.4)
I will advise or recommend self-medication to others.	34 (16.7)	57 (28.1)	58 (28.6)	34 (16.7)	20 (9.9)
I have confidence in my ability to manage my illness.	29 (14.3)	55 (27.1)	69 (34.0)	35 (17.2)	15 (7.4)
I believe that I can diagnose my health condition.	9 (4.4)	24 (11.8)	58 (28.6)	64 (31.5)	48 (23.6)
I believe that there is no training needed to start self-medication practice.	7 (3.4)	18 (8.9)	27 (13.3)	68 (33.5)	83 (40.9)
I believe that easy access to healthcare information and facilities is the main cause of self-medication practice.	42 (20.7)	96 (47.3)	44 (21.7)	12 (5.9)	9 (4.4)
The availability of OTC medicines and the belief in its safety leads me to practice self-medication	38 (18.7)	92 (45.3)	56 (27.6)	11 (5.4)	6 (3.0)
I can diagnose different diseases because I am a pharmacy student	14 (6.9)	44 (21.7)	66 (32.5)	39 (19.2)	40 (19.7)
I can treat different diseases because I am a pharmacy student.	14 (6.9)	35 (17.2)	62 (30.5)	44 (21.7)	48 (23.6)
Attitude score, median (IQR)	31.0 (7.1)				

IQR: interquartile range, *n*: frequency, N: sample size.

**Table 5:** Factors associated with the participants’ attitude about self-medication (N = 203).

Factors	Attitude Toward Self-Medication				Chi-Square (x^2^)	*p*-Value
	Positive, *n* (%)		Neutral, *n* (%)	Negative, *n* (%)		
Age	19–21	47 (31.5)	100 (67.1)	2 (1.3)	1.4	0.492
	22–25	14 (25.9)	40 (74.1)	0 (0.0)	1.2	0.563
Sex	Male	16 (34.8)	30 (65.2)	0 (0.0)		
	Female	45 (28.7)	110 (70.1)	2 (1.3)		
Academic year	Year 1	15 (30.0)	35 (70.0)	0 (0.0)	3.9	0.696
	Year 2	21 (36.8)	35 (61.4)	1 (1.8)		
	Year 3	13 (25.0)	38 (73.1)	1 (1.9)		
	Year 4	12 (27.3)	32 (72.7)	0 (0.0)		
Ethnicity	Malay	33 (32.4)	69 (67.6)	0 (0.0)	3.5	0.745
	Chinese	24 (28.6)	58 (69.0)	2 (2.4)		
	Indian	2 (22.2)	7 (77.8)	0 (0.0)		
	Other	2 (25.0)	6 (75.0)	0 (0.0)		
Current residency	University housing	43 (28.7)	106 (70.7)	1 (0.7)	8.8	0.069
	With family	3 (27.3)	7 (63.6)	1 (9.1)		
	Private housing	15 (35.7)	27 (64.3)	0 (0.0)		
Family members working in the healthcare sector	No	24 (37.5)	40 (62.5)	0 (0.0)	3.2	0.200
	Yes	37 (26.6)	100 (71.9)	2 (1.4)		

*n*: frequency, N: sample size.

**Table 6:** Practice patterns of self-medication among pharmacy undergraduates (N = 203).

Question	Answer Options	*n* (%)
Within the last six [6] months, have you engaged in the practice of self-medication as defined in this survey?	Yes	133 (65.5)
	No	70 (34.5)
You answered “NO” for this question. What was your reason? *	Fear of using the wrong medication	38 (18.7)
	Fear of adverse effects of the medication	31 (15.3)
	Lack of knowledge and experience	39 (19.2)
	Lack of confidence to self-medicate	31 (15.3)
	Had a bad experience with past self-medication practice	3 (1.5)
	I had no illness in the specified time	41 (20.2)
	Other	0 (0)
You answered “YES” for this question. How often do you practice self-medication?	During the last month	53 (26.1)
	During the last three [3] months	47 (23.2)
	During the last six [6] months	33 (16.3)
What was your source of information about the medications? *	Healthcare professionals	100 (49.3)
	Experience from previous treatment	105 (51.7)
	Drug Reference Books (MIMS, BNF, Lexicomp, etc.)	53 (26.1)
	Friend/Relatives/Neighbours	50 (24.6)
	Internet	60 (30.0)
	Other	1 (0.5)
Where do you get the medications for self-medication? *	Retail community pharmacy	132 (65.0)
	Leftover from previous treatment	60 (30.0)
	From family members/friends/neighbours	48 (23.7)
	Supermarket	16 (7.9)
	Internet/online store	15 (7.4)
	Other	0 (0)
How do you request the medications if the source of the medications is a retail community pharmacy? *	By mentioning the names of the medications	167 (82.3)
	By mentioning the signs and symptoms of illness	157 (77.4)
	By showing the medication container	79 (38.9)
	By showing a piece of paper on which, the names of the medications are written	44 (21.7)
	Other	0 (0)
Why do you practice/prefer self-medication? *	Time constraint	106 (52.2)
	Minor illness treatment	154 (75.9)
	Lack of confidence/trust in available healthcare services	10 (4.9)
	Emergency case	66 (32.5)
	Self-medication is cheaper	71 (35.0)
	I used the medication before	127 (62.6)
	I want to have experience with the medication/self-learning opportunity	23 (11.3)
	Other	0 (0)

*: Respondents could pick more than one answer (percentage summation ≠ 100%).

**Table 7:** Factors associated with the participants’ practice pattern concerning self-medication (N = 203).

Factors		Practicing Self-Medication Within the Last Six Months (*n*, %)	Do not Practice Self-Medication Within the Last Six Months (*n*, %)	Chi-Square (x^2^)	*p*-Value
Sex	Male	29 (63.0)	17 (37.0)	0.16	0.688
	Female	104 (66.2)	53 (33.8)		
Age	19–21	91 (61.6)	58 (38.9)	4.9	0.027 *
	22–25	42 (77.8)	12 (22.2)		
Academic year	Year 1	22 (44.0)	28 (56.0)	15.8	0.001 *
	Year 2	42 (73.7)	15 (26.3)		
	Year 3	34 (65.4)	18 (34.6)		
	Year 4	35 (79.5)	9 (20.5)		
Ethnicity	Malay	70 (68.6)	32 (31.4)	1.0	0.811
	Chinese	52 (61.9)	32 (38.1)		
	Indian	6 (66.7)	3 (33.3)		
	Other	5 (62.5)	3 (37.5)		
Residency	University housing	93 (62.0)	57 (38.0)	3.2	0.203
	With family	8 (72.7)	3 (27.3)		
	Private housing	32 (76.2)	10 (23.8)		
Family members working in the healthcare sector	Yes	47 (73.4)	17 (26.6)	2.6	0.107
	No	86 (61.9)	53 (38.1)		

*: The chi-square statistic is significant at the 0.05 level.

**Table 8:** Factors associated with the participants’ practice pattern about self-medication using multiple logistic regression (N = 203).

Variable	Multiple Logistic Regression		
	Model 1		
	β	OR ^a^ (95% CI)	*p*-Value
Age			
19–21	Reference		
22–25	0.278	1.321 (0.400–4.359)	0.648
Sex			
Female	Reference		
Male	−0.199	0.820 (0.396–1.698)	0.593
Academic Year			
Year 1	Reference		
Year 2	1.249	3.487 (1.543–7.879)	0.003
Year 3	0.817	2.263 (0.987–5.190)	0.054
Year 4	1.355	3.878 (0.929–16.191)	0.063

^a^ Variable selection using the backward (LR) method. Multicollinearity and interaction terms were checked, and the Hosmer–Lemeshow test was not found (*p*-value = 0.427).

## Data Availability

All data are available in the manuscript.

## Supplementary Materials

The following supporting information can be downloaded at: https://www.mdpi.com/article/10.3390/pharmacy13030079/s1, Table S1: Questionnaire of the study.
