# Supplementary material for: Knowledge, Attitudes, and Practices Toward Self-Medication Among Pharmacy Undergraduates in Penang, Malaysia: A Cross-Sectional Study
Source: Pharmacy (Basel). 2025 Jun 2;13(3):79. doi: 10.3390/pharmacy13030079 (PMC12197184; doi:10.3390/pharmacy13030079)
Supplement: Supplementary file 1 [file pharmacy-13-00079-s001.zip › pharmacy-3638917-supplementary.pdf]

Table S1. Questionnaire of the study

Knowledge, attitudes, and practices toward self-medication among pharmacy undergraduates in Penang, Malaysia: A cross-sectional study

Part I: Socio-demographic data

1- Age in years
19-21
22-25
>25

2- Gender
Male
Female

3- Academic year
Year 1
Year 2
Year 3
Year 4

4-Marital status
Single
Married
Divorced
Separated
Widowed

5-Ethinicity
Malay
Chinese
Indian
Other

6- Study status
Full time student
Part time student

7- Current residency
University housing
Private housing
With family

8- Family members working in the healthcare sector
Yes
No

Part II: Knowledge of participants about appropriate self-medication practice

In this section, we would like to access your knowledge regarding appropriate self-medication practices, emphasizing responsible usage of medications, please answer by Yes or No

**Questions**	**Answer Options**

Do you know that some medications cannot be taken with other medications?	○ Yes	○ No
Do you know that some medications cannot be taken with alcoholic drinks?	○ Yes	○ No
Do you know that some medications cannot be taken with certain foods?	○ Yes	○ No
Do you know that some medications are contraindicated or cannot be given to children?	○ Yes	○ No
Do you know that some medications are contraindicated or cannot be given when pregnant?	○ Yes	○ No
Do you know that some medications are contraindicated or cannot be given when breastfeeding?	○ Yes	○ No
Do you know that some medications are contraindicated or cannot be given to people with chronic illnesses?	○ Yes	○ No
Did you stop taking your medications without consulting with a healthcare professional for confirmation or guidance?	○ Yes	○ No
Do you know that certain medications cannot be shared with family members, friends, neighbours, etc.?	○ Yes	○ No
Do you check the expiry date of the medications before purchasing/before use?	○ Yes	○ No

Part III: Questions related to the attitude of participants towards self-medication.

In this section, we are interested to know about your attitudes towards self-medication. Please indicate how much you agree with each of the following statements.

1 = Strongly disagree, 2 = Disagree, 3 = Neutral, 4 = Agree, 5 = Strongly agree

**Statements**	**Answer Options**				
I believe self-medication is a part of self-care.	○ 1	○ 2	○ 3	○ 4	○ 5
I would like to start/continue my self-medication therapy.	○ 1	○ 2	○ 3	○ 4	○ 5
I will advise or recommend self-medication to others.	○ 1	○ 2	○ 3	○ 4	○ 5
I have confidence in my ability to manage my illness.	○ 1	○ 2	○ 3	○ 4	○ 5
I believe that I can diagnose my health condition.	○ 1	○ 2	○ 3	○ 4	○ 5
I believe that there is no training needed to start self-medication practice.	○ 1	○ 2	○ 3	○ 4	○ 5

I believe that easy access to healthcare information and facilities is the main cause of self-medication practice.	○ 1 ○ 2 ○ 3 ○ 4 ○ 5
The availability of OTC medicines and the belief in its safety leads me to practice self-medication	○ 1 ○ 2 ○ 3 ○ 4 ○ 5
I can diagnose different diseases because I am a pharmacy student	○ 1 ○ 2 ○ 3 ○ 4 ○ 5
I can treat different diseases because I am a pharmacy student.	○ 1 ○ 2 ○ 3 ○ 4 ○ 5

Part IV: Questions related to self-medication practices

The term "self-medication" in the context of this survey refers to health-related behaviors undertaken by individuals based on self-decisions concerning the use of Over-the-Counter

(OTC) Medications, Non-Prescription Drugs, Supplements, Leftover Drugs, or Medications Taken/Shared from Friends/Relatives.

For the purposes of this study, the usage of prescription medications and medications taken under the suggestion or guidance of a healthcare professional is not considered as self-medication practice. In this section, we are interested to know about your self medication practices (*select all applies)

Question	Answer Options
Within the last six (6) months, have you engaged in the practice of self-medication as defined in this survey?	Yes
	No
You answered "NO" for this question. What was your reason? *	Fear of using the wrong medication
	Fear of adverse effects of the medication
	Lack of knowledge and experience
	Lack of confidence to self-medicate
	Had a bad experience with past self-medication practice
	I had no illness in the specified time
	Other
You answered "YES" for this question. How often do you practice self-medication?	During the last month
	During the last three (3) months
	During the last six (6) months
What was your source of information about the medications? *	Healthcare professionals
	Experience from previous treatment
	Drug Reference Books (MIMS, BNF, Lexicomp, etc)
	Friend/Relatives/Neighbours
	Internet

	Other
Where do you get the medications for self-medication? *	Retail community pharmacy
	Leftover from previous treatment
	From family members/friends/neighbours
	Supermarket
	Internet/online store
	Other
How do you request the medications if the source of the medications is a retail community pharmacy? *	By mentioning the names of the medications
	By mentioning the signs and symptoms of illness
	By showing the medication container
	By showing a piece of paper on which, the names of the medications are written
	Other
Why do you practice/prefer self-medication? *	Time constraint
	Minor illness treatment
	Lack of confidence/trust in available healthcare services
	Emergency case
	Self-medication is cheaper
	I used the medication before
	I want to have experience with the medication/self-learning opportunity
	Other
What was the health condition that led you to practice self-medication?*	Headache
	Fever Cough/Cold/Flu
	Sore throat
	Ulcer in mouth
	Muscle pain/muscle ache
	Rash/allergies
	Toothache
	Stomach pain
	Diarrhea
	Period cramp/menstruation pain
	Dry eyes
	Constipation
	Other
What are the medications that you used for self-medication purposes?*	Pain reliever/Fever reducer
	Flu and Cold relief/Allergy medication
	Blocked-nose relief/Nasal decongestant

	Cough relief
	Stomach ache reliever
	Constipation relief/laxative
	Anti-diarrheal
	Antiulcer
	Eye drop/Artificial tear
	Muscle and joint pain relief
	Skin moisturizer cream/ointment
	Anti-itch cream/ointment
	Others

This is the end of the questionnaires

Thanks to your contribution to the self-medication research!
